# Adrenal (Pro)renin Receptor Expression and Serum Soluble (Pro)renin Receptor Concentration in Primary Aldosteronism

**DOI:** 10.1155/2020/9640103

**Published:** 2020-09-28

**Authors:** Daisuke Watanabe, Satoshi Morimoto, Noriko Morishima, Yoichiro Kato, Yoji Nagashima, Noriyuki Shibata, Atsuhiro Ichihara

**Affiliations:** ^1^Department of Endocrinology and Hypertension, Tokyo Women's Medical University, 8-1 Kawada-Cho, Shinjuku-Ku, Tokyo 162-8666, Japan; ^2^Department of Pathology, Tokyo Women's Medical University, 8-1 Kawada-Cho, Shinjuku-Ku, Tokyo 162-8666, Japan; ^3^Department of Surgical Pathology, Tokyo Women's Medical University, 8-1 Kawada-Cho, Shinjuku-Ku, Tokyo 162-8666, Japan

## Abstract

The (pro)renin receptor [(P)RR] is a multifunctioning protein playing roles in various pathological conditions. A soluble form of (P)RR [s(P)RR] has been considered a biomarker for (P)RR expression in tissues. Expression of (P)RR has been described in aldosterone-producing adenoma (APA), but the roles of (P)RR have yet to be fully determined. This study investigated the significance of (P)RR and serum s(P)RR concentrations in patients with APA. We evaluated associations between (P)RR expression and expression of CYP11B2, an aldosterone synthase, and aldosterone production by the adrenal glands and assessed the relationships between serum s(P)RR concentration and background factors. (P)RR colocalized with CYP11B2 and expression levels of (P)RR were positively associated with those of CYP11B2 in APA tissues. (P)RR immunoreactivity in these tissues correlated positively with plasma aldosterone concentrations (PAC) and urinary aldosterone excretion. Also, in APA, (P)RR mRNA abundance was positively correlated with *β*-catenin mRNA abundance. Significant positive correlations were identified between serum s(P)RR concentration and plasma glucose, hemoglobin A1c, and serum creatinine levels, but not with PAC (in either peripheral vein or adrenal vein) or adrenal (P)RR expression level. This study showed that (P)RR expression level correlates with CYP11B2 expression in APA tissues and PAC and urinary aldosterone excretion, suggesting that (P)RR expression may contribute to aldosterone synthesis via CYP11B2 activation in APAs, although serum s(P)RR concentration failed to show any significant relationship with adrenal (P)RR expression. Adrenal (P)RR activity might offer a therapeutic target in the treatment of PA, although this issue needs to be investigated in future studies.

## 1. Introduction

Primary aldosteronism (PA) was first described as a clinical syndrome characterized by hypokalemia, hypertension, and impaired glucose tolerance by Conn in 1955 [1]. PA is defined as autonomous aldosterone production leading to a downregulation of renin and is characterized by histological features including adrenal aldosterone-producing adenoma (APA) and idiopathic hyperaldosteronism. This pathology represents the most common endocrine form of secondary hypertension and its prevalence has been increasing recently. The incidence of cardiovascular morbidity is well known to be higher in patients with PA than in patients with essential hypertension [2].

CYP11B2 is one of the enzymes that play an essential role in aldosterone synthesis and are involved in the pathogenesis of APA [3]. Nishimoto et al. demonstrated the presence of CYP11B2 in APA using a specific antibody against CYP11B2 [4]. Recent studies have demonstrated that both CYP11B2 expression in the tumor and tumor size contribute to the overproduction of aldosterone in APA, and CYP11B2 immunohistochemical staining is thus considered a useful histopathological tool to confirm excessive aldosterone production [5].

The (pro)renin receptor [(P)RR] was cloned and identified in 2002 as a single transmembrane domain protein that binds to both renin and prorenin [6]. Widely distributed in important organs and tissues such as the kidneys, heart, brain, liver, placenta, and pancreas, (P)RR is thought to be a multifunctioning protein. (P)RR binds to vacuolar H^+^–adenosine triphosphatase (V-ATPase) and low-density lipoprotein receptor-related protein 6 to form a Wnt signaling receptor complex as an adaptor protein, showing that (P)RR is essential for normal Wnt signal transduction [7]. While Wnt signaling plays a crucial role in pattern formation during embryogenesis, previous reports have demonstrated that aberrant Wnt/*β*-catenin signaling is associated with APA development and controls aldosterone production [8]. Yamamoto et al. recently demonstrated that (P)RR is expressed in human adrenocortical tumors, including APA, although the mechanisms responsible for regulating (P)RR in APA have not yet been fully explored [9].

(P)RR exists in three different molecular forms: a full-length transmembrane protein; a soluble form of the (pro)renin receptor [s(P)RR]; and a truncated form comprising the transmembrane and cytoplasmic domains. Full-length (P)RR undergoes protease-mediated cleavage to produce the N- and C-terminal fragments, with the N-terminal fragment secreted extracellularly as s(P)RR. Blood concentrations of s(P)RR have been considered as a biomarker reflecting the activity of (P)RR in tissues [10, 11], and increased s(P)RR concentrations have been demonstrated in patients with chronic kidney disease (CKD) [12], hypertension [10], heart failure [13], and preeclampsia [14]. We have also reported that serum s(P)RR level was independently associated with organ damage, including arteriosclerosis and renal dysfunction, in patients with PA, suggesting that serum s(P)RR level could be used as a biomarker for increased risk of organ damage in these patients [15]. However, whether serum s(P)RR level is associated with (P)RR expression in APA has not been clarified.

On the basis of these background findings, this study was conducted to (1) evaluate the association of (P)RR expression with the expression of CYP11B2 and aldosterone production in the adrenal glands and (2) assess the relationships between serum s(P)RR concentration and background factors, including (P)RR expression and aldosterone production in the adrenal glands.

## 2. Materials and Methods

### 2.1. Study Population and Design

This study enrolled 64 patients who had been diagnosed with unilateral PA at Tokyo Women's Medical University (TWMU) Hospital between May 2012 and June 2019. The study protocol was approved by the ethics committee of TWMU Hospital. Consent has been obtained from each patient or subject after a full explanation of the purpose and nature of all procedures used.

All patients were diagnosed with PA according to the guidelines of the Japan Endocrine Society, including case detection, confirmatory tests, and subtype classification [16]. Three confirmatory loading tests for the diagnosis of PA were performed: the captopril challenge test, the furosemide upright test, and the saline infusion test. All patients showed positive results in at least two of these three tests. To avoid interfering with the renin-angiotensin-aldosterone system, these patients were treated with only calcium channel blockers (cilnidipine was excluded due to its aldosterone-lowering effects [17]) and *a*-blocker during the workup for PA. After confirmatory loading tests, adrenal venous sampling (AVS) was performed in all patients for subtype classification. The adrenal venous blood aldosterone/cortisol (A/C) ratio after cosyntropin stimulation was calculated bilaterally. In AVS, the selectivity index (SI), lateralized ratio (LR), and contralateral ratio (CR) were used. SI was defined as cortisol_adrenal vein_/cortisol_inferior vena cava_. Adrenal vein cannulation was considered successful if the SI was >5 or cortisol in the adrenal vein was >200 *μ*g/dL. LR was defined as (A/C_adrenal vein_)/(A/C_contralateral adrenal vein_), and CR was defined as (A/C_contralateral adrenal vein_)/(A/C_inferior vena cava_). All patients showed LR > 4.0 and were finally diagnosed with unilateral PA.

After the diagnosis of unilateral PA, 51 patients underwent laparoscopic adrenalectomy of the dominant side based on AVS results. Before adrenalectomy, CT was performed using a commercially available scanner to analyze the adrenal gland in contiguous 1.0 mm thick slices. Nonionic iodinated contrast agent was administered intravenously in a routine manner. All patients showed unilateral adrenal adenoma on CT images. The maximum area of each adrenal tumor was calculated from CT images using ImageJ version 1.52p (National Institutes of Health, Bethesda, MD).

### 2.2. Background Factors

At enrollment, information was collected on sex, age, and body mass index (BMI). Office blood pressure (BP) and pulse rate (PR) were measured with the patient in a sitting position after resting for at least 5 min. The first readings at each visit were used for this study.

### 2.3. Blood and Urinary Examinations

Plasma potassium, creatinine, uric acid, cholesterol, glucose (PG), and hemoglobin A1c (HbA1c) levels were measured using standard methods. Homeostasis model assessment (HOMA) was used as a measure of insulin resistance (HOMA-R = insulin (*μ*U/mL) × glucose (mg/dL)/405)) and *ß*-cell function (HOMA-*β* = 360 × insulin (*μ*U/mL)/(glucose (mg/dL)—63)) [18]. Plasma renin activity (PRA), plasma aldosterone concentration (PAC), and 24 h urinary aldosterone excretion (U-Aldo) were measured at an external laboratory (SRL Inc., Tokyo, Japan). PRA and PAC from venous blood samples were measured with the patient in a sitting position (for at least 15 min) at the first admission. Serum s(P)RR level was measured, in both peripheral and adrenal venous blood samples, using an enzyme-linked immunosorbent assay (ELISA) kit consisting of a solid-phase Sandwich ELISA with two kinds of highly specific antibody [19]. The 24 h urine collection was obtained while the patients were consuming their usual diet and without changing their level of physical activity.

### 2.4. Adrenal Tumors

Adrenal tissues were obtained at surgery from patients with APA. Immediately after surgical removal, tissues were fixed in 10% formalin and embedded in paraffin. Resected adrenal tumors were assessed microscopically, and specimens were pathologically confirmed as adrenal adenoma. For immunohistochemical staining analysis, the paraffin-embedded section (4 *μ*m thick) was deparaffinized and incubated overnight at 4°C with polyclonal anti-rabbit (P)RR antiserum (dilution 1 : 200) [19], which had been raised in a rabbit by injecting the peptide fragment of human (P)RR corresponding to 222–239 amino acids (human (P)RR 222–239, custom synthesis; Immuno-Biological Laboratories, Fujioka, Japan), or polyclonal anti-rabbit CYP11B2 antiserum (gifted from K. Nishimoto, Department of Uro-Oncology, Saitama Medical University International Medical Center, Japan; dilution 1 : 100) [4]. These sections were then incubated for 30 min at room temperature with a secondary antibody labeled with rhodamine for (P)RR and FITC for CYP11B2 staining. Staining with 4′,6-diamidino-2-phenylindole (DAPI) was used to visualized nuclei. At the same time, (P)RR and CYP11B2 were also identified, followed by the Dako REAL™ EnVision™ Detection System (Dako, Glostrup, Denmark). For immunohistochemistry, sections incubated with solutions from which the primary antibodies were omitted served as negative reaction controls. These results were evaluated for the following semiquantitative analysis of immunostaining.

### 2.5. Quantitative Real-Time RT-PCR (qPCR)

Total RNA was extracted from APA using TRI REAGENT (Molecular Research Center, Cincinnati, OH) according to the manufacturer's instruction. cDNA was synthesized by using a High Capacity cDNA Reverse Transcription Kit (Thermo Fisher Scientific, Waltham, MA). The qRT-PCR was performed on a QuantStudio 3 Real-Time PCR System (Thermo Fisher Scientific). The human (P)RR primer sequences were 5′ AGA TGA CAT GTA CAG TCA TTA TGG TGG 3' (forward) and 5′ TGC TGG GTT CTT CCC TTG T 3' (reverse). The primer of *β*-catenin (Assay ID: Hs00355045_m1) was obtained from Thermo Fisher Scientific. GAPDH was taken as endogenous controls and the relative expressions were calculated via the 2-ΔΔCt method.

### 2.6. Western Blot Analysis

Adrenal fresh frozen tissues (10–50 *μ*g) were homogenized using BioMasher II^®^ (Nippi, Tokyo, Japan) with RIPA buffer (Sigma, St. Louis, MO). Extracted proteins were loaded on any kD™ Mini-PROTEIN TGX gels (Bio-Rad, Hercules, CA) transferred to PVDF membranes and detected using the ECL method. The membrane was incubated overnight with polyclonal anti-rabbit (P)RR antiserum (dilution 1 : 100) and/or a polyclonal anti-rabbit CYP11B2 antiserum (dilution 1 : 100). Expression of glyceraldehyde-3-phosphate dehydrogenase (GAPDH) was studied as an internal control using anti-GAPDH monoclonal antibody (Proteintech, Chicago, IL; dilution 1 : 5000). For immunoblotting, blots incubated with solutions from which the primary antibodies were omitted served as negative reaction controls. Protein levels were quantified using ImageJ version 1.52p.

### 2.7. Semiquantitative Analysis of Immunostaining

To investigate the functional significance of the immunostaining results, we evaluated the immunohistochemical intensity of (P)RR and CYP11B2 in each adrenal tumor using ImageJ color deconvolution plugin [20, 21]. We used these calculated results as the DAB staining intensity (DAB score) of (P)RR and CYP11B2.

### 2.8. Statistical Analysis

All data are expressed as mean ± SD or median (interquartile range). Differences in adrenal venous s(P)RR levels between dominant and nondominant adrenal sides were analyzed using the Mann–Whitney *U* test. Univariate correlations were determined by calculating Spearman rank correlation coefficients. Values of *p* < 0.05 were considered significant. All analyses were performed using JMP version 14 (SAS Institute Inc., Cary, NC).

## 3. Results

Among 51 postoperative patients, paraffin-embedded specimens were obtained and subjected to immunohistochemical analysis in 44 patients with APA (termed Group A, 23 males; 52.3%). The preoperative clinical and endocrinological characteristics of Group A are shown in Table 1. Mean systolic and diastolic blood pressure was slightly elevated. Forty patients (91%) were hypokalemic (serum potassium < 3.5 mEq/L). Hormonal data revealed suppressed PRA and increased PAC. These laboratory data were compatible with PA due to APA. CT findings indicated a median maximum tumor area of 62.0 mm^2^ (range, 43.9–85.9 mm^2^). In AVS results, after cosyntropin stimulation, median LR was 14.4 (range, 7.5–22.0), and CR suppression was recognized in 42 of the 44 patients (95%).

### 3.1. Histological Findings, mRNA, and Protein Expression in the Investigated APA

**Figure 1:**
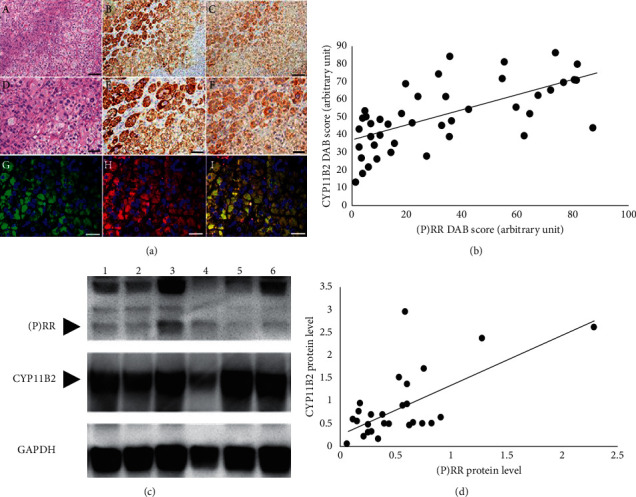
(a) Histological findings in aldosterone-producing adenoma. A and D: hematoxylin and eosin staining; B and E: immunohistochemical staining for CYP11B2; C and F: immunohistochemical staining for (P)RR; G: immunofluorescent staining for CYP11B2 (green) and DAPI (blue); H: immunofluorescent staining for (P)RR (red) and DAPI (blue); and I: immunofluorescence-merged images showing dual-labeled CYP11B2 and (P)RR (yellow) and DAPI (blue). Bar lengths: (A–C) 100 *μ*m; (D–F) 50 *μ*m; and (G–I) 20 *μ*m. (P)RR: (pro)renin receptor and DAPI: 4′,6-diamidino-2-phenylindole. (b) Scatter plots showing the positive association between adrenal (P)RR and CYP11B2 expression. (c) Western blot analysis in aldosterone-producing adenoma. The intensity of bands for (P)RR and CYP11B2 is shown in each lane (depicted by a black arrow). The other bands are nonspecific bands. (d) Scatter plots showing the positive association between adrenal (P)RR and CYP11B2 protein levels measured by Western blotting results. (P)RR: (pro)renin receptor and GAPDH: glyceraldehyde 3-phosphate dehydrogenase.

Histological images are shown in Figure 1(a). In Group A patients, hematoxylin and eosin staining revealed that the tumor mainly comprised clear cells with lipid-rich cytoplasm and eosinophilic compact cells mixed with a small number of compact cells (Figure 1(a):A, D).

We subsequently analyzed whether CYP11B2 or (P)RR protein is expressed in human APA using immunohistochemical analysis. CYP11B2 expression was heterogeneously immunolocalized throughout the tumor area (Figure 1(a):B, E). In APAs, intense (P)RR immunostaining was found in compact cells with dominant granular eosinophilic cytoplasm (Figure 1(a):C, F). Colocalization of (P)RR and CYP11B2 in APA was studied by immunofluorescence staining. (P)RR was colocalized with CYP11B2 in the same cells (Figure 1(a):G, H, I). DAB score of (P)RR also showed a significant positive correlation to that of CYP11B2 in APA tissue (rs = 0.66, *p* < 0.05) (Figure 1(b)).

**Figure 2:**
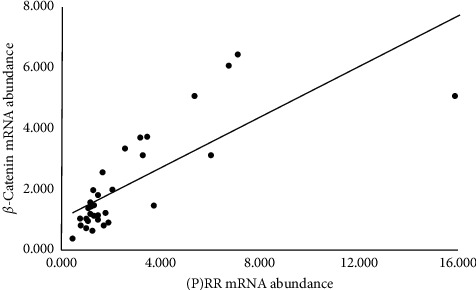
Scatter plots showing the positive association between adrenal (P)RR and *β*-catenin mRNA abundance. (P)RR: (pro)renin receptor.

Among 51 postoperative patients, adrenal fresh frozen tissues were also obtained from 32 patients. We further quantitatively analyzed the correlation between (P)RR and CYP11B2 protein abundance in APA tissues using Western blotting. The antibody raised against (P)RR and CYP11B2 gave a signal with a molecular mass of around 35 kDa and 48.5 kDa, respectively, in APA tissues (Figure 1(c)). The intensity of bands for (P)RR and CYP11B2 was calculated and (P)RR protein abundance correlated significantly with CYP11B2 protein abundance (rs = 0.41, *p* < 0.05) (Figure 1(d)). Next, we analyzed the correlation between (P)RR and *β*-catenin mRNA levels in APA frozen samples using real-time RT-PCR methods. (P)RR mRNA abundance was significantly correlated with *β*-catenin mRNA abundance (rs = 0.78, *p* < 0.05) (Figure 2).

### 3.2. Hormonal and Immunohistochemical Findings

**Figure 3:**
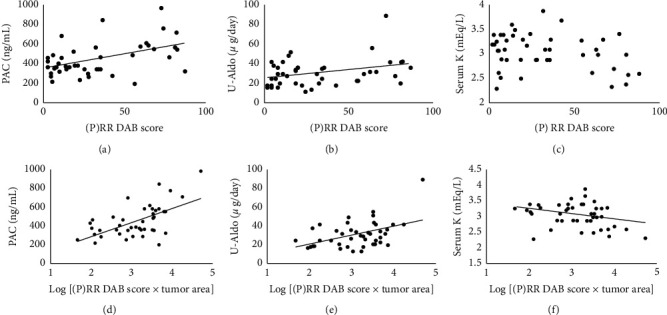
Correlation of (P)RR expression both before and after adjusting tumor area with PAC, U-aldo, and serum K. (P)RR: (pro)renin receptor; PAC: plasma aldosterone concentration; U-aldo: urinary aldosterone concentration; and K: potassium.

To determine whether expression of CYP11B2 or (P)RR in APA correlated with hormonal activities, associations of adrenal (P)RR expression levels with PAC, U-Aldo, and serum potassium level were studied in Group A patients. CYP11B2 DAB score multiplied by tumor area correlated positively with PAC and U-Aldo and negatively with serum potassium level (Table 2). (P)RR DAB score correlated positively with PAC and U-Aldo (rs = 0.42, *p* < 0.05; rs = 0.32, *p* < 0.05, respectively). In addition, (P)RR DAB score multiplied by tumor area correlated positively with PAC and U-Aldo (rs = 0.49, *p* < 0.05; rs = 0.36, *p* < 0.05, respectively) and negatively with serum potassium level (rs = −0.32, *p* < 0.05) (Figure 3).

### 3.3. Serum s(P)RR Levels

In Group A patients, the serum s(P)RR levels were not different between male (23.2 ± 6.0 ng/mL, *n* = 23) and female (21.5 ± 6.6 ng/mL, *n* = 21) patients. Serum creatinine levels and eGFR showed positive and negative correlations with s(P)RR, respectively. In addition, PG, HbA1c, and HOMA-R revealed significant positive correlations with serum s(P)RR levels. However, no significant correlations were seen between s(P)RR levels and any of age, BMI, blood pressure, laboratory data (lipid parameters and serum potassium level), endocrinological data (PRA, PAC, and U-Aldo), or immunoreactivity of CYP11B2 and adrenal (P)RR (Table 3).

**Figure 4:**
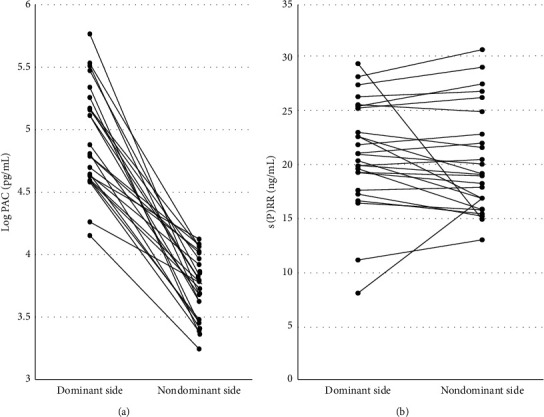
PAC and s(P)RR levels. Blood samples were collected from both dominant- and nondominant-side adrenal veins. (P)RR: (pro)renin receptor and PAC: plasma aldosterone concentration.

During AVS, we collected blood samples from bilateral adrenal veins in 25 patients of all 64 patients. PAC or serum s(P)RR levels were compared between adrenal veins on the dominant and nondominant sides. PAC was clearly elevated on the dominant side (*p* < 0.05), but s(P)RR level showed no significant differences between both sides (Figure 4).

## 4. Discussion

We demonstrated here that (P)RR expression is observed in human APA tissues using immunohistochemical staining methods. The immunohistochemical results clearly demonstrated the presence of parenchymal cells co-expressing (P)RR and CYP11B2 in the tumor lesion of APA. To the best of our knowledge, this is the first report to evaluate the relationships among expression of adrenal (P)RR, CYP11B2, and PAC in patients with APA. The present study demonstrated two major findings. First, a significant positive correlation was identified between the immunohistochemical intensity of (P)RR and CYP11B2, and (P)RR DAB score correlated positively with PACs and U-Aldo. Second, in patients with APA, s(P)RR levels correlated negatively with eGFR and positively with PG, HbA1c, and HOMA-R but were not associated with peripheral or adrenal venous PACs, degree of adrenal (P)RR, or CYP11B2 expression. These data suggest that (P)RR may contribute to aldosterone synthesis through CYP11B2 activation in APAs, although serum s(P)RR concentration failed to show a significant relationship with adrenal (P)RR expression.

The present study demonstrated that, similar to the results of a previous study [5], both PACs and urinary aldosterone excretion correlated significantly with immunohistochemical intensity of CYP11B2 multiplied by tumor area. Of particular interest, our study found a significant positive correlation between DAB scores of (P)RR and CYP11B2 in APA tissues. CYP11B2, which catalyzes the final steps of aldosterone biosynthesis, is reportedly deeply involved in the pathogenesis of APA [3]. These facts may suggest that (P)RR, as well as CYP11B2, is a determinant of tumor aldosterone production and clinical manifestations of hyperaldosteronism in APA.

(P)RR is a single transmembrane domain receptor that mediates renin and prorenin specific effects. Binding of renin and prorenin to the (P)RR increases their enzymatic activity and directly triggers intracellular signal transduction, resulting in accelerated cell proliferation and production of profibrotic gene expressions, by a mechanism independent of angiotensin II generation [22]. Recently, (P)RR has also been shown to be indispensable for the integrity of V-ATPase and to act as an adaptor between V-ATPase and the Wnt signaling pathway [7]. The Wnt signaling pathway, through *β*-catenin signaling, is also important for the normal development and maintenance of the zona glomerulosa within the adrenal cortex and is associated with the development of APA [23]. In addition, *β*-catenin is known to play an essential role in the control of basal and angiotensin II-induced aldosterone secretion, by activating angiotensin II type 1 receptor (AT1R), CYP21 and CYP11B2 transcription [8]. These findings may therefore indicate that (P)RR plays a crucial role in the CYP11B2 transcription through activation of the Wnt/*β*-catenin signaling pathway. On the other hand, a previous *in vitro* study using HAC15 adrenocortical cells demonstrated that the binding of prorenin to (P)RR triggers the generation of angiotensin I from angiotensinogen, ensuring the formation of angiotensin II and activation of the AT1R leading to transcription of CYP11B2 and amplification of aldosterone production [24]. These findings and our immunohistochemical staining results raised the possibility that (P)RR plays an important role in the regulation and/or production of aldosterone through activation of CYP11B2 transcription in human APA tissues. Pharmacological inhibition of Wnt/*β*-catenin signaling, such as administration of anti-(P)RR neutralizing antibody and/or inhibitors of Wnt/*β*-catenin signaling, might have therapeutic potential to attenuate the (P)RR-induced aldosterone hypersecretion in APA, although this possibility needs to be investigated in future studies.

Since s(P)RR is generated by intracellular protease-mediated cleavage of full-length (P)RR, we hypothesize that circulating s(P)RR level may be related to increased intra-adrenal (P)RR activity, consequently inducing an increase in CYP11B2 transcription and aldosterone production in APA. However, we demonstrated that, in the setting of PA, serum s(P)RR levels had no significant correlation with PRA in peripheral veins, PAC in peripheral or adrenal veins, or adrenal (P)RR expression. From these results, we speculate that serum s(P)RR levels are not influenced by (P)RR expression in “small” adrenal glands and are influenced by (P)RR expression in organs other than the adrenal gland. In fact, s(P)RR level is reportedly elevated under certain pathological conditions, especially in cases of CKD [12, 25]. In human kidney with CKD, reduced renal excretion of s(P)RR and increased intra-adrenal (P)RR expression are speculated to contribute to the elevation of s(P)RR. Another report suggested that (P)RR might be involved in impaired insulin secretion by causing *β*-cell dysfunction in the pancreas [26]. Intracellular mitogen-activated protein kinase cascade activated by (P)RR is considered to regulate *β*-cell function and to contribute to the pathogenesis of glucose intolerance. In addition, (P)RR itself plays a critical role in the development of diabetes through V-ATPase-linked function in pancreatic *β*-cells [27]. Although the significance of s(P)RR in certain pathophysiological circumstances remains elusive, our data suggested that s(P)RR concentration is associated with the development of renal dysfunction and worsening glucose metabolism in patients with APA. On the basis of these findings, in patients with APA, (P)RR activity in kidney and pancreatic *β*-cells seems to influence the elevation of circulating s(P)RR levels. In further research, pathophysiological results of s(P)RR would be helpful to identify the novel mechanisms of renal function and glucose metabolism in patients with APA.

## 5. Limitations

Some limitations to this study need to be considered when interpreting the results. First, we could not clearly investigate the pathophysiological effects of (P)RR on CYP11B2 transcription *in vitro*. Besides (P)RR-Wnt/*β*-catenin signaling, other factors triggered by (P)RR activation might influence CYP11B2 transcription in APA tissues. Second, only patients with APA detectable on CT were investigated in this study. (P)RR may demonstrate unprecedented effects on tumorigenesis and/or aldosterone biosynthesis in cases of microadenoma not undetectable on CT. Third, we did not evaluate (P)RR activity in organs other than the adrenal glands in patients with APA. Kidney, pancreatic, and skeletal (P)RR activity may play pathophysiological roles in the development of CKD and diabetes in patients with APA.

## 6. Conclusion

We showed that (P)RR expression in APAs correlated with PAC, raising the possibility that (P)RR may contribute to aldosterone synthesis in APAs. Modulation of adrenal (P)RR activity might represent a therapeutic target for the treatment of PA and might contribute to reducing the cerebrocardiovascular morbidity and mortality associated with autonomous aldosterone hypersecretion. Increased serum s(P)RR concentration was associated with renal dysfunction and impaired glucose metabolism in patients with APA but was not correlated with circulating PAC, suggesting that serum s(P)RR concentration may be influenced by secretion of s(P)RR from organs outside the adrenal glands. Further studies are needed to investigate the mechanisms by which serum s(P)RR concentration is regulated in patients with APA.

## Figures and Tables

**Table 1:** Clinical characteristics of pathologically confirmed aldosterone-producing adenoma group.

Characteristics	
*n*	44
Age	50.7 ± 11.3
Gender (male/female)	23/21
Body mass index (kg/m^2^)	23.4 ± 4.1
Blood pressure (mmHg)	
Systolic	140.5 ± 16.9
Diastolic	88.3 ± 11.8
Laboratory data	
Creatinine (mg/dL)	0.77 ± 0.21
eGFR (mL/min/1.73 m^2^)	78.5 ± 19.0
Serum potassium (mEq/L)	3.1 ± 0.4
LDL-cholesterol (mg/dL)	118.0 ± 30.4
HDL-cholesterol (mg/dL)	58.7 ± 17.5
Triglyceride (mg/dL)	101 (72.3–155.8)
Plasma glucose (mg/dL)	96.5 (88.3–114.8)
Hemoglobin A1c (%)	5.5 (5.3–6.1)
HOMA-R	1.2 (0.8–1.9)
HOMA-*β*	50.1 (38.0–78.4)
Hormonal data	
PAC (pg/mL)	387 (347.8–549.5)
PRA (ng/ml/h)	0.3 (0.2–0.5)
U-aldo (*μ*g/day)	29 (20–38)
Adenoma size (mm^2^)	62.0 (43.9–85.9)
Lateralization ratio in AVS after cosyntropin loading	14.4 (7.5–22.0)
Contralateral suppression (yes/no)	(42/2)

PRA: plasma renin activity; PAC: plasma aldosterone concentration; U-aldo: urinary aldosterone excretion; and AVS: adrenal venous sampling. Data are expressed as mean ± SD or median (25^th^–75^th^).

**Table 2:** Single regression analyses with CYP11B2 expression multiplied by tumor area.

	rs	*p* value
Plasma aldosterone concentration (pg/mL)	0.47	0.001
24-hour urinary aldosterone excretion (*μ*g/day)	0.33	0.028
Serum potassium level (mEq/L)	−0.33	0.029

**Table 3:** Single correlation analyses with serum soluble (pro)renin receptor levels in the pathologically confirmed aldosterone-producing adenoma group.

	rs	*p* value
Age	0.181	0.239
Body mass index (kg/m^2^)	0.220	0.151
Blood pressure (mmHg)		
Systolic	−0.092	0.551
Diastolic	0.009	0.954
Laboratory data		
Creatinine (mg/dL)	0.385	0.009
eGFR (mL/min/1.73 m^2^)	−0.368	0.014
LDL-cholesterol (mg/dL)	0.085	0.581
HDL-cholesterol (mg/dL)	−0.193	0.210
Triglyceride (mg/dL)	0.125	0.419
Serum potassium (mEq/L)	0.038	0.806
Plasma glucose (mg/dL)	0.382	0.011
Hemoglobin A1c (%)	0.420	0.005
HOMA-R	0.399	0.007
HOMA-*β*	−0.038	0.805
Hormonal data		
PRA (ng/ml/h)	0.129	0.405
PAC (pg/mL)	−0.033	0.830
U-aldo (*μ*g/day)	0.231	0.131
Immunohistochemical findings		
CYP11B2	0.096	0.535
CYP11B2 multiplied by tumor area	−0.020	0.897
(P)RR	0.111	0.469
(P)RR multiplied by tumor area	0.039	0.800

eGFR: estimated glomerular filtration rate; LDL: low-density lipoprotein; HDL: high-density lipoprotein; HOMA-R: homeostasis model assessment for insulin resistance; HOMA-*β*: homeostasis model assessment of *β*-cell function; PRA: plasma renin activity; PAC: plasma aldosterone concentration; and U-aldo: urinary aldosterone excretion.

## Data Availability

All data generated or analyzed during this study are included within the article.
